# Development and psychometric analysis of the student–teacher relationship scale – short form

**DOI:** 10.3389/fpsyg.2015.00898

**Published:** 2015-06-26

**Authors:** Michele Settanni, Claudio Longobardi, Erica Sclavo, Michela Fraire, Laura E. Prino

**Affiliations:** Department of Psychology, University of TurinTurin, Italy

**Keywords:** teacher–child relationship, Rasch analysis, STRS, educational psychology, validity

## Abstract

The purpose of this study is the construction and validation of an Italian Short Form version of the Student–Teacher Relationship Scale (STRS; Fraire et al., 2013). The analyses were conducted on 1256 students and 210 teachers. The STRS is a self-report measure assessing teachers’ perception of the quality of their relationship with students ranging from preschool to third grade. The items were selected from the original Italian adaptation of the regular STRS (Pianta, 2001) through Rasch (1960/1980) analysis, which allowed us to identify a subset of items with proven psychometric properties. The STRS-SF consists of two subscales: Conflict (eight items) and Closeness (six items). Results indicate that the 14-item instrument shows good internal consistency (α>0.80), high correlations with the scales from the regular STRS (*r* > 0.90) and equivalence across gender.

## Introduction

Student–teacher relationships are micro systems that consist of the multiple interrelated perceptions that both parties have about their interactions (Pianta et al., 2003). Said perceptions are personal representations imbued with feelings, evaluations, beliefs, and expectations, and they are important because they are real, from a psychological standpoint, and they have the power to influence the behavior of each party significantly (Stuhlman and Pianta, 2002).

It has been often stated that an educational model oriented toward a conception of the ecological development of the individual cannot help but consider the varied forms of interaction and interchange that children experience both inside and outside schools (Bronfenbrenner and Morris, 2006; Tudge et al., 2009; Hamre and Pianta, 2010). Therefore, it follows quite naturally that teacher–student interactions should be measured in order to examine their nature, and make valid explanations regarding their influence on children’s development. Several national regulations concerning the education system have underlined the value of the student–teacher relationship. For example, in Italy, the “Nuovi Orientamenti” [“New Orientations”] decree (Decreto Ministeriale, 1991), has underlined the role that adult relational competencies have a fundamental value in establishing a functional relationship that considers the affective, cognitive, and social aspects of the educational practice.

During the last decade, most research on teacher–pupil relationships has utilized the Student–Teacher Relationship Scale (STRS; Pianta, 2001) in some manner. The instrument assesses three elements: Closeness, Conflict and Dependency, which define the behavior patterns that characterize the relationship between teacher and pupil (Pianta, 1994; Birch and Ladd, 1997). These dimensions are consistent across child age, ethnicity, and socioeconomic status; they remain stable from kindergarten to secondary school. More precisely, they constitute a sort of conceptual map of relationship perception (Lynch and Cicchetti, 1992).

As described by Birch and Ladd (1997), *Closeness* is a warm affective relationship with a teacher, capable of promoting positive attitudes toward school, open communication, involvement, and engagement. Students that display Closeness tend to use the teacher as a resource for facing and overcoming their problems; they are also more inclined to share their own emotions and experiences, especially in moments of strife/discomfort (e.g., Pianta et al., 2003). Finally, greater Closeness may encourage children’s learning and school performance and is associated with more positive feelings about school (e.g., Birch and Ladd, 1997), fewer behavioral problems, more behavioral competencies, and social skills (e.g., Pianta and Stuhlman, 2004; Buyse et al., 2008).

The *Conflict* dimension measures the negative aspects in the relationship, such as discordant interactions and the absence of a satisfying teacher–pupil relationship. These aspects act as important stress factors for students in a school setting; student–teacher conflicts constitute a situation of tension and favor negative behaviors. Especially during primary school, conflictual relations with the teacher are at the base of many behavioral problems. They jeopardize social abilities and interactions (Mantzicopoulos, 2005; Doumen et al., 2008); impede good school performance (DiLalla et al., 2004); obstruct the development of positive attitudes toward schoolwork (Hamre and Pianta, 2007) and increase the risk of regular absences from school.

Finally, the *Dependency* dimension measures possessive, “clingy” behavior and subjectiveness of the child in relation with the teacher. A child that depends on a teacher in an excessive manner tends to inhibit his or her behaviors and, consequently, hesitates in exploring the class/school environment. The child’s sticky behavior poses an obstacle to normal social interaction with peers, favoring feelings of solitude and a negative attitude toward school in general (Birch and Ladd, 1997). Dependency has not received as much attention as the other two dimensions, in the research on student–teacher relationships.

The 28 item version of the STRS is rated on a five point Likert-type scale. Previous validity studies have reported sufficient internal consistency, thus validating the scale as an effective and reliable measure for the teacher’s perception of the relationship with his or her pupils. However, there are few studies conducted on the first version of the STRS (Pianta and Steinberg, 1992; Saft, unpublished; Steinberg, unpublished) and only Webb and Neuharth-Pritchett (2011) have examined the factorial validity confirmative of the current 28 item US version, reaching the conclusion that a 26 item version was an effective and reliable measure for studying the teacher–pupil relationship. In a similar manner, Gregoriadis and Tsigilis (2008) have examined the applicability of the STRS in the Greek educational setting with an exploratory factor analysis (EFA) and have obtained the same results as Webb and Neuharth-Pritchett (2011). In the Netherlands, Koomen et al. (2012) have examined the applicability of the STRS using a confirmatory factor analysis (CFA). Their results confirm the validity of the instrument in the Dutch school system, with the only difference of the age extension up to 12 years. On the other hand, in the Italian context, there have been relevant contributions made by Fraire et al. (2008, 2013), and Molinari and Melotti (2010). Recently, Tsigilis and Gregoriadis (2008) have validated a Greek short form of the STRS, and initial psychometric studies show that it is a promising instrument in terms of construct validity and ease of administration in a sample. Considering these recent research results, our study focuses on the development of an Italian short form of the STRS (Pianta, 2001). To reach this aim, we conducted our analyses on data from 210 teachers that responded to the items of the already validated and translated Italian STRS full version (Fraire et al., 2008, 2013). To validate the short form, we tested its psychometric properties to verify the absence of significant changes on instrument reliability and validity with respect to the original version. The development of a short STRS is a useful tool for researchers and teachers low on time (Ang, 2005), because of its reduced administration times and lean question list. Having both versions validated and available for the Italian context will allow researchers to choose the one that best suits the applicational or research aims established.

## Materials and Methods

### Participants

The total number of teachers involved in the study is 210. The sample is not sex-balanced (92% females), reflecting the current Italian gender distribution for kindergarten and elementary schools teachers (Organization for Economic Co-operation and Development [OECD], 2011). The age distribution and the years of experience (see **Table 1**) of the sample mirror the characteristics of the population of teachers in the Italian territory. The majority of the teachers involved in the study (57%) had spent more than 15 h per week in the classroom from which the children were selected; all the teachers had worked with the class since the beginning of the school year.

**Table 1:** Distributions of type of teacher characteristics.

		Percentage
Gender	Female	91.5
	Male	8.5
Age (years)	18–30	3.8
	31–40	24.4
	41–50	42.1
	More than 50	29.8
Teaching experience (years)	Less than 1	0.9
	1–5	7.0
	6–10	12.3
	11–15	17.1
	16–20	16.7
	21–25	11.5
	26–30	14.3
	31–35	13.8
	More than 35	6.4

The final completed questionnaires collected data referring to 1256 children, aged 3–9 years (*M* = 6.0, SD = 1.6). The sample was balanced for gender (males = 50.5%). Forty-four percent (*n* = 550) of the sample attended preschool, and the remaining 56% (*n* = 706) attended the first 3 years of elementary school.

### Instruments

#### Student–Teacher Relationship Scale (STRS; Pianta, 2001)

The Student–Teacher Relationship Scale is a self-report instrument consisting of 28 items developed with reference to the Attachment Theory, especially the Attachment Q-set (Waters and Deane, 1985). It is designed to be used with children aged 3–8 (preschool through third grade; e.g., Howes and Ritchie, 1999). Items are evaluated on a five-point Likert scale, ranging from 1 (definitely does not apply) to 5 (definitely applies). The final form of the scale presents three factors, identified as Conflict, Closeness, and Dependency subscales. The original instrument by Pianta (2001) has been adapted and validated to the Italian context (Fraire et al., 2013). The adapted version of the instrument consists of 22 items that strengthens the psychometric basis of the STRS by confirming the three-dimensional structure of the original instrument: Closeness (α = 0.86), Conflict (α = 0.91), and Dependency (α = 0.69).

#### Academic Performance and Commitment

Teachers, in addition to filling out the STRS questionnaire, specified the child’s age and gender, and evaluated his or her academic achievement and commitment on a five-point Likert scale. They were also asked to provide information concerning their own sociodemographic statuses and teaching experience.

### Procedure

From each of the different areas in Italy (Northwest, Northeast, Center, South and Islands), we randomly selected five schools. All participants accepted to take part in the study after having been presented with a detailed description of the research. The study was carried out in accordance with the Italian Law and with the norms of the Code of Ethics of the Italian Psychology Association (AIP, 2000).

#### Data Analysis Strategy

The development of a short form of the instrument was based on the results of the analyses conducted on the original version. With the aim of creating a shorter version of the STRS we decided, on the basis of theoretical considerations and accordance to the international literature on STRS short forms (Gregoriadis and Tsigilis, 2008), to leave out of the instrument the items composing the Dependency subscale. In order to reduce the number of items we based our procedure on Rasch model. One of the most relevant characteristic of this measurement model over the classical test theory (CTT) is the possibility to transform raw ordinal variable scales into interval variable scales, having the log-odds unit (or *logit*) as unit of measurement. The rationale for the choice of this analytic approach is that Rasch (1960/1980) analysis permits to assess both monodimensionality of the scales and measurement invariance of the single items. Hence, given a larger set of items, employing the Rasch model allows to identify a smaller core set of items with proven psychometric characteristics (Clark et al., 1983; Thomas, 2011). For these reasons, Rasch models have been employed successfully by many authors for both item selection and shortening of questionnaires (e.g., Nijsten et al., 2006; Goetz et al., 2013; Balsamo et al., 2014).

As a result, we conducted a series of Rasch (1960/1980) analyses on the Closeness and Conflict subscales to evaluate their items’ performance and gage the possibility of eliminating them. In selecting the items suitable for removal, we considered two different criteria: item fit and measurement invariance. Concerning fit statistics, items were considered for removal when they did not show sufficient compliance to the Rasch model as valued through Infit and Outfit statistics, which are the mean square fit statistics most commonly used by scholars (Linacre, 2008).

The expected value of fit statistics is 1, its interval ranging from 0 to infinity. When values are lower than 1 we witness an absence of stochasticity, as expected in data. Values higher than 1 indicate possible violations of the one-dimensionality premise; values located between 0.6 and 1.3 are considered acceptable (Wright and Linacre, 1994). As a second step, we judged subscale item measurement invariance using Differential Item Functioning (DIF). DIF allows us to examine if the items included in an instrument have a significantly different mean – even though it shows equal levels of the trait studied (in this case Closeness and Conflict) – for the groups considered inside the population studied (Bond and Fox, 2007). In this study, we evaluated the DIF for both school level (preschool vs. primary school) and gender (males vs. females). The DIF was evaluated by comparing the estimates of the item measures for every subgroup contemplated, and confronting the latter through *t*-test, as suggested by Linacre (2008).

Rasch (1960/1980) analysis has been employed in an iterative way: candidate items were deleted one at a time. After each item deletion the item scale parameters, fit and DIF statistics were recomputed. When more than one item were congruent with removal criteria, the one with less desirable characteristics was removed first and the analysis was redone.

As a final passage, the adequacy of the instrument’s reduction process was evaluated by correlating the scores of each subscale before and after reduction, and by computing the intercorrelation and reliability coefficients, and the correlations with other study variables.

## Results

### Rasch Analysis

Monodimensionality being a requisite for the employment of Rasch model analyses (Rasch, 1960/1980), both the Conflict and Closeness subscale were analyzed separately. In both cases the analysis was based on the whole sample (*N* = 1256) and the Partial Credit model (PCM) was used (Masters, 1982).

#### Conflict Subscale (STRS-SF)

The results of the first analysis conducted on the original 10 item subscale are displayed in **Table 2** (left side). Regarding the items, **Table 2** shows that none of the 10 items exhibited fit issues. Item Infit and Outfit are inside the acceptance interval (0.60–0.30), therefore none of the items had to be removed for poor fit. During the second step of the item removal procedure, measurement invariance was verified by referring to the DIF for data concerning the children’s school level and gender. Overall, DIF analysis allowed us to identify only two problematic elements (all the other items showed non-significant results). Item Number 16 (20 of the US version) proved to be significantly more difficult for females [females: measure = -0.16 logit; males: measure = -0.41 logit; *t*(1254) = 3.10, *p* = 0.001]. Item Number 17 (22 of the US version) also exhibits a problematic DIF, contemplating both school level and gender. This item proved to be easier for preschool students [preschool: measure = 0.33 logit; elementary school: measure = -0.30 logit; *t*(1254) = 2.79, *p* = 0.005; females: measure = -0.33 logit; males: measure = -0.51 logit; *t*(1254) = 2.35, *p* = 0.019]. Based on these results, we removed both items from the subscale. A second analysis conducted on the remaining 8 items showed adequate fit (see **Table 2**, right side) and no statistically significant DIF for any item. The measure range for the items of the reduced Conflict subscale proved to be quite limited (-0.64, -0.42), but not different from the one calculated for the original scale (-0.73, -0.33).

**Table 2:** Rasch analysis: item measure, SE, fit statistics, and item-total correlations of the Conflict subscale.

	Original version (10 item)					Shortened version (8 items)				
Item	Measure	SE	Infit	Outfit	*r*	Measure	SE	Infit	Outfit	*r*
2	0.42	0.05	1.02	1.07	0.58	0.33	0.05	1.00	1.03	0.59
9	0.33	0.05	0.97	0.93	0.60	0.24	0.05	0.94	0.92	0.62
10	0.11	0.04	0.99	0.99	0.64	0.02	0.04	0.93	0.88	0.67
13	0.20	0.05	1.07	1.14	0.62	0.11	0.05	1.00	1.03	0.65
15	-0.64	-0.04	1.13	1.17	0.72	-0.73	0.04	1.15	1.12	0.74
16	-0.30	0.04	0.87	0.93	0.68					
17	-0.40	0.04	0.91	0.94	0.70					
18	0.05	0.04	1.06	1.14	0.62	-0.04	0.04	1.03	1.07	0.64
19	0.10	0.04	0.95	1.03	0.61	0.01	0.04	0.95	0.97	0.62
20	0.14	0.04	1.15	1.19	0.61	0.05	0.04	1.09	1.14	0.64

*N = 1256. r’s are item-total correlations*.

#### Closeness Subscale (STRS-SF)

For the Closeness subscale, we used the same analysis procedure employed for the Conflict subscale. Results of the original item analysis are displayed in **Table 3**. As highlighted, all items showed rather elevated correlation coefficients when compared to the total (48, -0.71). However, two items (Numbers 3 and 5 – 3 and 7 in the original version) manifested excessively high levels of Infit and Outfit. Therefore, we removed both items from the subscale, one at the time, while rechecking the fit statistics. After removal, the analysis was once again conducted on the remaining six items, which did non-exhibit additional fitting problems (see **Table 3**). Concerning measurement invariance, the six items did not display a significant DIF for either school level or gender. The measure range for the items of the Closeness subscale also proved to be quite contained (-0.45, -0.51) when compared to the one calculated on the original items (-0.69, -1.13): this is owed to the exclusion of item Number three from the short form of the subscale, since it proved to be the most “difficult,” namely the one with the highest measure.

**Table 3:** Rasch analysis: item measure, SE, fit statistics, and item-total correlations of the Closeness subscale.

	Original version (eight item)					Shortened version (six items)				
Item	Measure	SE	Infit	Outfit	*r*	Measure	SE	Infit	Outfit	*r*
1	-0.46	0.04	0.87	0.80	0.66	-0.45	0.05	1.01	0.97	0.69
3	1.13	0.04	1.35	1.47	0.66					
4	-0.69	0.05	0.85	0.81	0.66	-0.72	0.05	1.01	1.00	0.69
5	-0.67	0.05	1.27	1.34	0.48					
7	0.13	0.04	0.94	0.95	0.67	0.23	0.04	1.08	1.09	0.71
12	0.58	0.04	0.98	1.05	0.70	0.79	0.04	1.04	1.11	0.76
21	0.18	0.04	0.82	0.79	0.71	0.3	0.04	0.87	0.84	0.75
22	-0.19	0.04	0.93	0.95	0.65	-0.15	0.05	0.96	0.97	0.71

*N = 1256. r’s are item-total correlations*.

### Reliability and Correlations with Other Variables of Study

Adequacy to the reduction process operated was examined in two phases. First, the internal consistency of the subscales was considered, before and after item removal, along with its intercorrelation with other subscales. Subsequently, the subscale correlations with other variables (e.g., academic performance, commitment, school grade, and gender) were evaluated. **Table 4** displays the results of the analysis. One may notice the similarities between the scale means and the reliability coefficients of the original and short form versions of the subscales. Similarly, the correlations between the Closeness and Conflict subscales showed a small change, moving from -0.33 (for the original subscales) to -0.36 (for the short form subscales). Therefore, by confronting the Italian Long and Short form versions of the STRS, it emerges that the Conflict and Closeness subscales have elevated correlations between versions (Conflict, *r* = 0.98; Closeness, *r* = 0.94).

**Table 4:** Internal consistency, subscale means, SD, and intercorrelations among Student–Teacher Relationship Scale (STRS) and STRS-short subscales.

Scale	Original version					Shortened version				
	α	Scale mean (SD)	1	2	3	α	Scale mean (SD)	1	2	3^a^
(1) Conflict	0.91	1.51 (0.73)	–	-0.33^∗∗^	0.42^∗∗^	0.88	1.48 (0.73)	–	-0.36^∗∗^	0.41^∗∗^
(2) Closeness	0.86	3.83 (0.68)	–	–	0.11^∗∗^	0.86	3.81 (0.71)	–	–	0.07^∗^
(3) Dependency	0.69	1.68 (0.78)	–	–	–	–	–	–	–	–

*N = 1256. ^a^Correlations of the Conflict and Closeness short version subscales with the original Dependency subscale. ^∗^p < 0.05; ^∗∗^p < 0.01*.

**Table 5** displays the correlation between the subscales (original and short form versions) and other variables of study. For the dichotomous variables School Grade and Gender, point-biserial correlations were reported. As it may be noted, no relevant difference has emerged.

**Table 5:** Intercorrelations among the STRS subscales and Academic achievement, Commitment, School level, and Gender (*N* = 1256).

Scale	Academic achievement	Committment	School level^a^	Gender^a^
(1) Conflict (original/short)	-0.25^∗^/-0.23^∗^	-0.34^∗^/-0.33	-0.23^∗^/-0.22^∗^	-0.11^∗^/-0.09^∗^
(2) Closeness (original/short)	0.32^∗^/0.35^∗^	0.40^∗^/0.41	<-0.01/<-0.01	0.12^∗^/0.11^∗^

*^a^Point-biserial correlations. ^∗^p < 0.01*.

## Discussion

The Short Form of the instrument is based on the 22-item version that has already been adapted to the Italian context (Fraire et al., 2013). Unlike the extended version, which is structured on three factors, the Short Form is based on two factors: Closeness and Conflict.

Statistical analyses conducted on both versions of the STRS have highlighted the instrument’s validity and its applicability in an Italian setting. The data similarities derived from the analyses of both instruments confirm their validity for both internal consistency and correlation between subscales and other variables considered in the study.

Concerning the items that make up the Conflict scale, the use of Rasch models has brought us to the exclusion of items number 16 (*Dealing with this child drains my energy*) and 17 (*When this child is in a bad mood, I know we’re in for a long and difficult day*), on the basis that they appear to be *easier* for the Male group and, concerning only item number 17, for the Kindergarten group. Furthermore, in the Italian context, gender differences reflect a cultural specificity (Saraceno, 2003; Saraceno and Naldini, 2007; Ruspini, 2009). With referral to the influence that the child’s mood has on his or her level of conflictuality, we acknowledge that stereotypes and expectations have an effect on the construct level that is perceived by males and females and, consequently, by children who attend kindergarten or primary school. The Conflict subscale now contains eight items that capture specific aspects of the relationship with the single pupil and have nothing to do with the teacher’s available work energies or the effects of conflict on his or her entire day. The Conflict scale concerns the teacher’s perception of single manifestations of rage, incomprehension and frustration acted out by the child, and his or her difficulties in defining, and managing the conflictual aspects of such a relationship.

In the Short Form, the Closeness subscale is made up of six items that qualify the teacher’s aspects of emotional Closeness, sharing, trust, and perception of self-efficacy in the relationship with the child. Rasch model analysis has highlighted the potential multidimensionality of items number 3 (*If upset, this child will seek comfort in me*), and 5 (*When I praise this child, he/she beams with pride*), and has brought us to the idea of eliminating them from the Short Form version of the STRS. The removal of these two items does not alter the validity of the Closeness scale, as they are both aspects that are not central to the construct and could well be related to other dimensions. They are, in fact, the only two items of the Closeness scale that take into consideration the child’s specific moods or emotional responses.

## Conclusion

Regarding the proposal of a short form of the instrument, it was possible to operate a relevant reduction of both the Closeness and Conflict subscales’ item number on the basis of theoretical considerations and empirical data analysis and still allow them to maintain adequate psychometric qualities.

The Short Form presented in our study is derived from the extended Italian version of the STRS, for the purpose of obtaining a useful and valid instrument for this specific context (Oyserman and Markus, 1993). Ang (2005) has emphasized how much a Short Form of the STRS would be useful for research purposes: the reduced number of items allows for a quicker and easier compilation and ensures that the questionnaire can be flanked to other instruments without resulting in an excessive amount of work for the participants.

For this reason, and in complete support of Ang’s (2005) statements, we believed that an Italian Short Form of the STRS needed to be created. In addition, its existence will allow Italian researchers to participate in multicultural study comparisons, and to share their data with other colleagues around the world, using the same, solid instrument.

Furthermore, the instrument is conceived for an age span that includes two distinct school grades: Preschool (3–5 years) and Primary School (starting from 6 years). During the last 20 years, the Italian Government has created a series of laws and measures aimed at strengthening the union and continuity between these grades from both an organizational and didactic point of view, through the creation of Comprehensive Institutes (L.97/1994 and L.111/2011) that unite all grades from Preschool to eighth grade. Simultaneously, a single educational path has been established for teachers of both preschool and primary school.

The government’s desire to develop a single course of studies for children during their most important period of development concretizes itself in the particular attention that is paid to the establishment of functional relationships inside the school system. The transition between school grades, such as Preschool and Primary School, has been emphasized (Ministero della Pubblica Istruzione, 2013). The STRS Short Form constitutes a resource for studies and researches aimed at monitoring and supporting relational processes in their becoming.

Unfortunately, our study did not take into consideration predictive and concurrent validity. More specifically, we believe that it would be important to also investigate children’s present and future scholastic abilities, temperamental traits, behavioral problems, emotional, and social capabilities, and peer relationships. Similarly, it would be important to take into account variables that concern teachers, such as their prior professional experience, personality features, relational capacities, perception of self-efficacy, and perceived stress level.

We would also like to point out the potential predictive validity of the measure when used in longitudinal studies on school success and adaptability to an educational context. For this reason, we believe that it would be interesting to conduct such studies to gain a better understanding of these aspects. It would also be worthwhile to investigate the bidirectional characteristics of the student–teacher relationship in order to retrieve further information on its nature. Finally, it would also be relevant to confront the evaluations gathered by both parties (i.e., students and teachers) with those made by an external observer for the purpose of gaining an additional point of view on the student–teacher relationship. We hope that all these issues might be addressed in future studies.

## Conflict of Interest Statement

The authors declare that the research was conducted in the absence of any commercial or financial relationships that could be construed as a potential conflict of interest.
